# Risk of uveitis among children with autoimmune diseases: a nationwide matched-cohort study of 3,643 cases

**DOI:** 10.3389/fimmu.2025.1717805

**Published:** 2025-12-04

**Authors:** De-Yi Liu, Hou-Ting Kuo, Bing-Qi Wu, Henry Bair, Peng-Tai Tien, Ning-Yi Hsia, Alan Y. Hsu, Yih‐Dih Cheng, Yow‐Wen Hsieh, Yu‐Han Huang, Sing‐Ting Wang, Chun-Ju Lin, Chang-Ching Wei, Chun-Ting Lai, Yi-Ching Shao, Chun-Chi Chiang, Hsin Tseng, Huan-Sheng Chen, Yi-Yu Tsai

**Affiliations:** 1 Department of Ophthalmology, China Medical University Hospital, China Medical University, Taichung, Taiwan; 2 Department of General Medicine, China Medical University Hospital, Taichung, Taiwan; 3 Wills Eye Hospital, Philadelphia, PA, United States; 4 School of Medicine, College of Medicine, China Medical University, Taichung, Taiwan; 5 Department of Optometry, Asia University, Taichung, Taiwan; 6 Department of Pharmacy, China Medical University Hospital, Taichung, Taiwan; 7 School of Pharmacy, China Medical University, Taichung, Taiwan; 8 Department of Management Office for Health Data, China Medical University Hospital, Taichung, Taiwan; 9 College of Medicine, China Medical University, Taichung, Taiwan; 10 Division of Hematology and Oncology, Department of Internal Medicine, China Medical University Hospital, Taichung, Taiwan; 11 Division of Allergy, Immunology and Rheumatology, Department of Pediatrics, Children’s Hospital, China Medical University Hospital, Taichung, Taiwan; 12 Graduate Institute of Biomedical Sciences, College of Medicine, China Medical University, Taichung, Taiwan; 13 An‐Shin Dialysis Center, Excelsior Renal Service Co. Ltd. Taiwan Branch, Taichung, Taiwan

**Keywords:** pediatric uveitis, autoimmune diseases, diabetes mellitus, lipid metabolism, corticosteroids, immunosuppressive therapy and sulfasalazine

## Abstract

**Background:**

Pediatric uveitis, though accounting for less than 10% of all uveitis cases, presents significant diagnostic and therapeutic challenges due to its asymptomatic onset and potential for severe, vision-threatening complications. Despite known associations with autoimmune diseases, data on risk factors in Asian pediatric populations remain limited. This study aimed to quantify the risk of uveitis in Taiwanese children with autoimmune diseases, identify key comorbidities, and evaluate the effects of immunosuppressive therapies.

**Methods:**

Using Taiwan’s National Health Insurance Research Database (2009–2019), we conducted a nationwide retrospective cohort study of 3,643 pediatric patients with autoimmune diseases matched 1:1 to controls. Patients were followed for up to 12 years, with uveitis risk assessed through Cox proportional hazards models and cumulative incidence analyzed using Kaplan-Meier curves.

**Results:**

During a mean follow-up of 5.5 years, autoimmune diseases were associated with increased uveitis risk (adjusted HR [aHR] = 2.65 [95% CI, 1.67-4.19]), with juvenile idiopathic arthritis showing the highest risk (aHR = 25.70 [95% CI, 7.41-89.22]). Risk was significant only in adolescents aged 10-14 years (aHR = 2.58 [95% CI, 1.29-5.14]) and 15-18 years (aHR = 2.60 [95% CI, 1.27-5.31]) and was notably higher in patients without diabetes (aHR = 6.88 [95% CI, 2.54-18.61]) compared with those with diabetes (aHR = 1.67 [95% CI, 0.98-2.82]). In medication analysis, sulfasalazine use (aHR = 2.00 [95% CI, 1.04-3.84]) and high-daily dose prednisolone (≥30 mg/day; aHR = 2.25 [95% CI, 1.12-4.53]) were associated with increased risk, while moderate cumulative prednisolone doses were associated with a lower risk compared with low-dose exposure (aHR = 0.32 [95% CI, 0.13-0.79]).

**Conclusion:**

This cohort study identified distinct patterns of uveitis risk across specific autoimmune diseases and age groups. These findings suggest the need for risk-stratified ophthalmologic screening based on autoimmune diagnosis and age in pediatric patients requiring immunosuppressive therapy.

## 1 Introduction

Pediatric uveitis, though accounting for only 5-10% of all uveitis cases, represents a significant challenge in pediatric ophthalmology due to its potential for severe vision-threatening complications and substantial impact on healthcare systems (1–3). Global epidemiological data estimates an incidence of 4.3 per 100,000 children annually, with prevalence rates varying across different geographical regions and ethnicities (2, 4–6). Unlike adult uveitis, the pediatric form often presents asymptomatically, particularly in its early stages, creating a critical diagnostic challenge that is further complicated by difficulties in examining young children (7). This diagnostic complexity frequently results delay**s** treatment, increasing the risk of vision-threatening complications such as keratopathy, cataracts, and glaucoma. Up to 67% of cases developing complications if not managed appropriately (2, 3, 8). Given these significant risks and challenges in diagnosis, systematic screening protocols for high-risk pediatric populations are essential. However, unlike some Western countries guided by consensus recommendations (e.g., American College of Rheumatology) (9), Taiwan has not yet established a national or standardized pediatric uveitis screening protocol, highlighting the need for localized risk data. Early detection and timely intervention remain the cornerstone for preventing irreversible ocular damage and optimizing visual outcomes.

The pathogenesis of non-infectious pediatric uveitis involves complex interactions between genetic predisposition, immune dysregulation, and emerging concepts such as the gut microbiome and epigenetic modifications (10–13). Molecular studies have identified the central roles of autoreactive Th1 and Th17 cells, which, through interactions with antigen-presenting cells, drive the production of pro-inflammatory cytokines including IL-17A, TNF-α, and IFN-γ (14). The JAK-STAT signaling pathway and SOCS proteins serve as crucial immune regulators in both uveitis and associated autoimmune conditions (15).

While juvenile idiopathic arthritis (JIA) remains the most frequently identified systemic association in Western populations, the spectrum and prevalence of associated autoimmune conditions show notable geographical and ethnic variations (2, 8, 16, 17). Despite these expanding insights into immunopathogenesis, approximately 20-30% of pediatric uveitis cases remain idiopathic, with significant variations across different populations and ethnicities (18). These differences may partly be explained by population-specific genetic variations (e.g., prevalence differences in HLA alleles) and various environmental exposures, including infectious triggers and microbiome diversity (10, 19).

In Asian populations—particularly in Taiwan—comprehensive pediatric studies remain limited, despite the well-documented association between systemic autoimmune diseases and uveitis in adults (2). Given potential regional differences in autoimmune disease expression and uveitis presentation, population-specific studies are necessary. To address this gap, we conducted a nationwide retrospective cohort study to evaluate the risk of uveitis among children and adolescents with autoimmune diseases in Taiwan. Our findings provide insights into population-specific risk profiles and may inform the development of targeted screening strategies and personalized management protocols for pediatric uveitis.

## 2 Materials and methods

### 2.1 Study design and data source

We conducted a nationwide retrospective cohort study using Taiwan’s National Health Insurance Research Database (NHIRD) from 2009 to 2019 with follow-up through 2020. The NHIRD covers approximately 99.9% of Taiwan’s 23 million residents and contains comprehensive medical information including diagnoses, procedures, prescriptions, and healthcare utilization patterns. All diagnoses are coded according to the International Classification of Diseases (ICD-9/10) standards. The database undergoes regular auditing and validation by the National Health Insurance Administration to ensure data integrity. This study was approved by the Institutional Review Board of China Medical University Hospital (CMUH111-REC2-109(CR-1)), and the requirement for informed consent was waived due to the use of de-identified data.

### 2.2 Study population

We identified pediatric patients (age ≤18 years) with autoimmune diseases diagnosed between January 1, 2009, and December 31, 2019. Autoimmune diseases were defined by ICD-9/10 codes (**Supplementary Table 1**) and validated by the presence of a catastrophic illness certificate, which requires comprehensive clinical documentation and expert review for approval in Taiwan’s healthcare system. The catastrophic illness certificate requirement ensures diagnostic accuracy and minimizes misclassification bias. The approval date served as the index date for those patients.

To optimize statistical power (90% to detect a hazard ratio of 1.5 at α = 0.05), we applied 1:1 propensity score matching. Cases were matched to controls without autoimmune disease based on sex, age group, comorbidities, and index year. For the autoimmune group, the index date was defined as the date of catastrophic illness certificate approval. For controls, an index date was randomly assigned post-January 1, 2000, using a computer-generated sequence to reduce selection bias. Exclusion criteria are detailed in **Supplementary Method 1**. Briefly, patients with prior diagnoses of viral hepatitis, HIV, or tuberculosis were excluded due to potential confounding, as were those with missing demographic data.

### 2.3 Outcome and covariate

The primary outcome was defined as the first diagnosis of uveitis, identified using validated ICD-9-CM and ICD-10-CM codes (**Supplementary Table 2**).

Covariates included age, sex, comorbidities, and medication use. Given that epidemiologic data indicate distinct age distributions among JIA subtypes, we categorized patients into three age groups: <10, 10–14, and 15–18 years (20, 21). This classification is consistent with the Childhood Arthritis and Rheumatology Research Alliance (CARRA) consensus treatment plans, which reflect clinical practice by recognizing that pediatric rheumatology care often extends up to age 18, and occasionally to 19—beyond the stricter boundaries defined by ILAR criteria (22). Comorbidities included lipid metabolism disorders, hypertension, and diabetes mellitus, each defined by relevant ICD-9-CM and ICD-10-CM codes (**Supplementary Table 2**) prior to the index date.

Medication exposures were determined using Anatomical Therapeutic Chemical (ATC) codes for systemic corticosteroids and immunosuppressive agents, including prednisolone (H02AB06), cyclosporin (L04AD01), sulfasalazine (A07EC01), azathioprine (L04AX01), methotrexate (L04AX03), and mycophenolate (L04AA06), as detailed in **Supplementary Table 3**. Medication exposure was assessed as a time-dependent variable, defined as any prescription record from the index date until the primary outcome, disenrollment, or the end of the study period (December 31, 2020). For prednisolone, prescribed daily doses and cumulative doses were calculated from prescription records to evaluate potential dose-dependent effects.

### 2.4 Statistical analysis

Categorical variables were presented as numbers and percentages, while continuous variables were expressed as means and standard deviations (SD). The chi-square test was used to examine differences in categorical variables between the case and control groups, whereas the t-test was used for continuous variables. Additionally, the standardized mean difference (SMD) was calculated using Cohen’s d to assess differences between the two groups. An SMD of less than 0.1 indicated a negligible difference.

Uveitis incidence was calculated as events per 1,000 person-years. A multivariable Cox proportional hazards model was used to estimate the risk of uveitis for each variable, adjusting for gender, age group, comorbidities, and medication use. To account for polypharmacy (use of multiple concurrent medications), the medication exposure groups were not treated as mutually exclusive. Instead, the Cox models for each specific drug (e.g., methotrexate) were adjusted for concurrent exposure to the other studied immunosuppressive agents. Cumulative incidence was visualized using Kaplan–Meier curves, with group differences tested via the log-rank test. A two-sided p-value of <0.05 was considered statistically significant.

All statistical analyses were conducted using SAS software (version 9.4, SAS Institute Inc., Cary, NC), and graphs were generated using RStudio.

## 3 Results

### 3.1 Demographics of individuals with and without autoimmune disease

**Figure 1:**
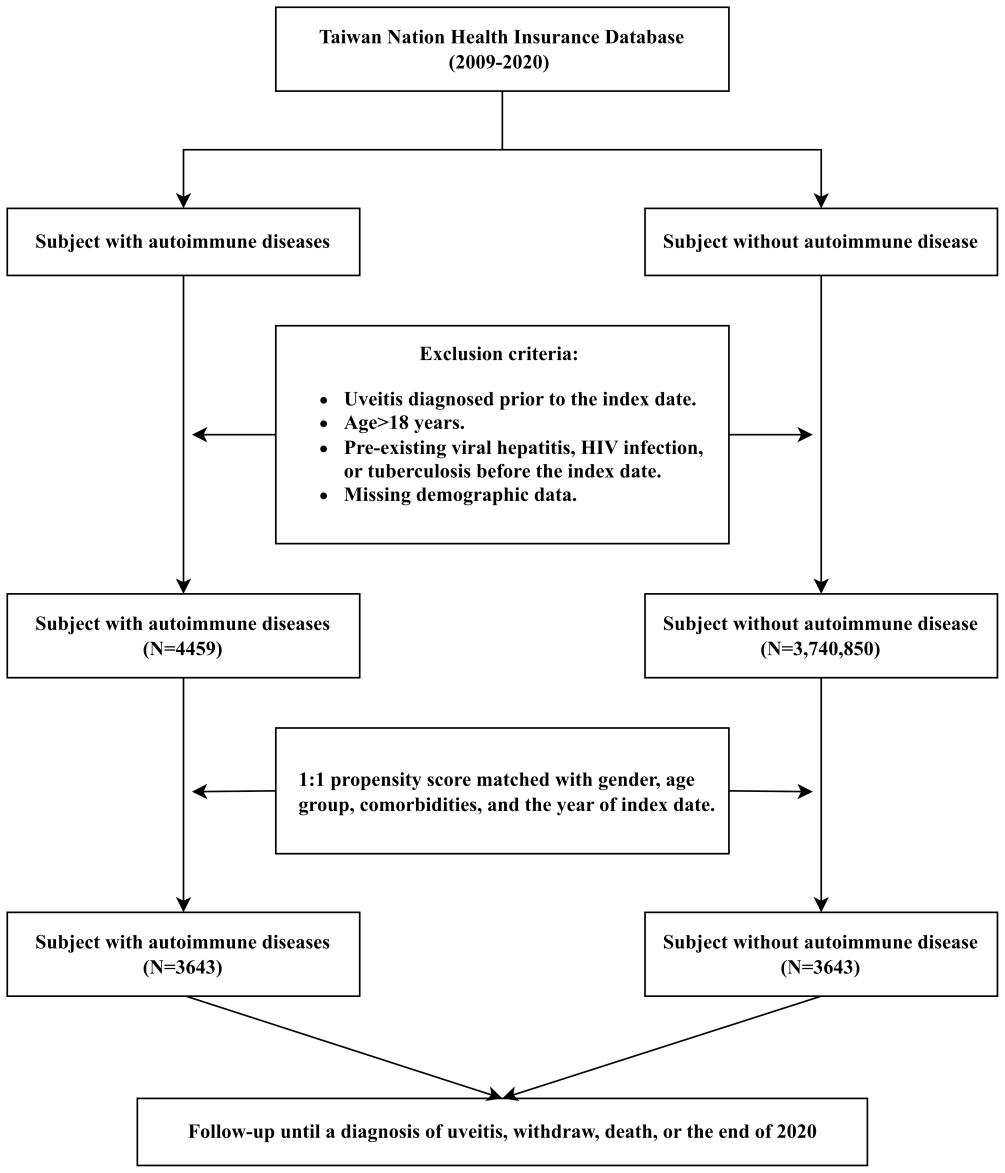
Flow diagram of participant selection and propensity score matching.This diagram illustrates the inclusion and exclusion process of subjects from the Taiwan National Health Insurance Research Database between January 1, 2009, and December 31, 2020. Patients with autoimmune diseases and matched controls without autoimmune disease were identified. Exclusion criteria included age older than 18 years, index year before 2009 or after 2019, prior diagnosis of uveitis, and history of viral hepatitis, HIV infection, or tuberculosis. Propensity score matching (1:1) was conducted based on sex, age groups, comorbidities, and index year.

After 1:1 propensity score matching, the study included 3,643 patients in both the autoimmune disease and control groups. The flowchart of patient selection is presented in **Figure 1**. The demographic and clinical characteristics were well-balanced between groups (standardized mean differences <0.1 for all variables, **Table 1**). The study population comprised 56.46% females in both groups (2,057 patients each), with age distribution as follows: <10 years (38.24% in the case group; 37.19% in the control group), 10–15 years (32.06% in the case group; 33.1% in the control group), and 15–18 years (29.7% in both groups). Diabetes mellitus was the most prevalent comorbidity (39.12% of total participants). The distribution of autoimmune diseases in the study group is presented in **Supplementary Table 4**, with type 1 diabetes mellitus (T1DM, 39.39%) being the most prevalent, followed by systemic lupus erythematosus (SLE, 20.2%).

**Table 1:** Baseline characteristics for individuals with and without autoimmune diseases.

	Before matching				After matching			
	Autoimmune diseases		P-Value	SMD	Autoimmune diseases		P-Value	SMD
Characteristics	No (n=3740850)	Yes (n=4459)			No (n=3643)	Yes (n=3643)		
Demographic Features								
Gender, No. (%)			<.001	—			>.99	—
Female	1766047 (47.21)	2580 (57.86)	—	0.22	2057 (56.46)	2057 (56.46)	—	<0.001
Male	1974803 (52.79)	1879 (42.14)	—	0.22	1586 (43.54)	1586 (43.54)	—	<0.001
Age, y, No. (%)			<.001	—			.57	—
<10	2178997 (58.25)	2161 (48.46)	—	0.02	1355 (37.19)	1393 (38.24)	—	0.02
10-14	775882 (20.74)	1216 (27.27)	—	0.15	1206 (33.10)	1168 (32.06)	—	0.02
15-18	785971 (21.01)	1082 (24.27)	—	0.08	1082 (29.70)	1082 (29.70)	—	<0.001
Age, mean (SD), y	8.76 (5.7)	10 (5.2)	<.001	0.28	11.06 (5.2)	10.99 (5.1)	.55	0.01
Comorbidities, No. (%)								
Lipid metabolism disorders	8471 (0.23)	282 (6.32)	<.001	0.35	252 (6.92)	173 (4.75)	<.001	0.09
Hypertension	2751 (0.07)	70 (1.57)	<.001	0.17	79 (2.17)	69 (1.89)	.41	0.02
Diabetes	2678 (0.07)	2241 (50.26)	<.001	1.42	1425 (39.12)	1425 (39.12)	>.99	<0.001
Medication, No. (%)								
Prednisolone	61195 (1.64)	1042 (23.37)	<.001	0.07	85 (2.33)	1031 (28.30)	<.001	0.77
Cyclosporin	1826 (0.05)	216 (4.84)	<.001	0.38	NA	216 (5.93)	<.001	0.41
Sulfasalazine	2289 (0.06)	317 (7.11)	<.001	0.39	NA	317 (8.70)	<.001	0.43
Azathioprine	2041 (0.05)	725 (16.26)	<.001	0.62	NA	725 (19.90)	<.001	0.69
Methotrexate	2614 (0.07)	587 (13.16)	<.001	0.55	NA	587 (16.11)	<.001	0.61
Mycophenolate	236 (0.01)	96 (2.15)	<.001	0.55	NA	96 (2.64)	<.001	0.61
Follow-up time, y								
Duration, mean (SD), y	6.50 (3.2)	5.90 (3.2)	<.001	0.19	5.68 (3.0)	5.50 (3.1)	.011	0.06

SD, Standard Deviation; SMD, Standardized Mean Difference.

— Indicates not applicable.

### 3.2 Risk factors for uveitis

**Figure 2:**
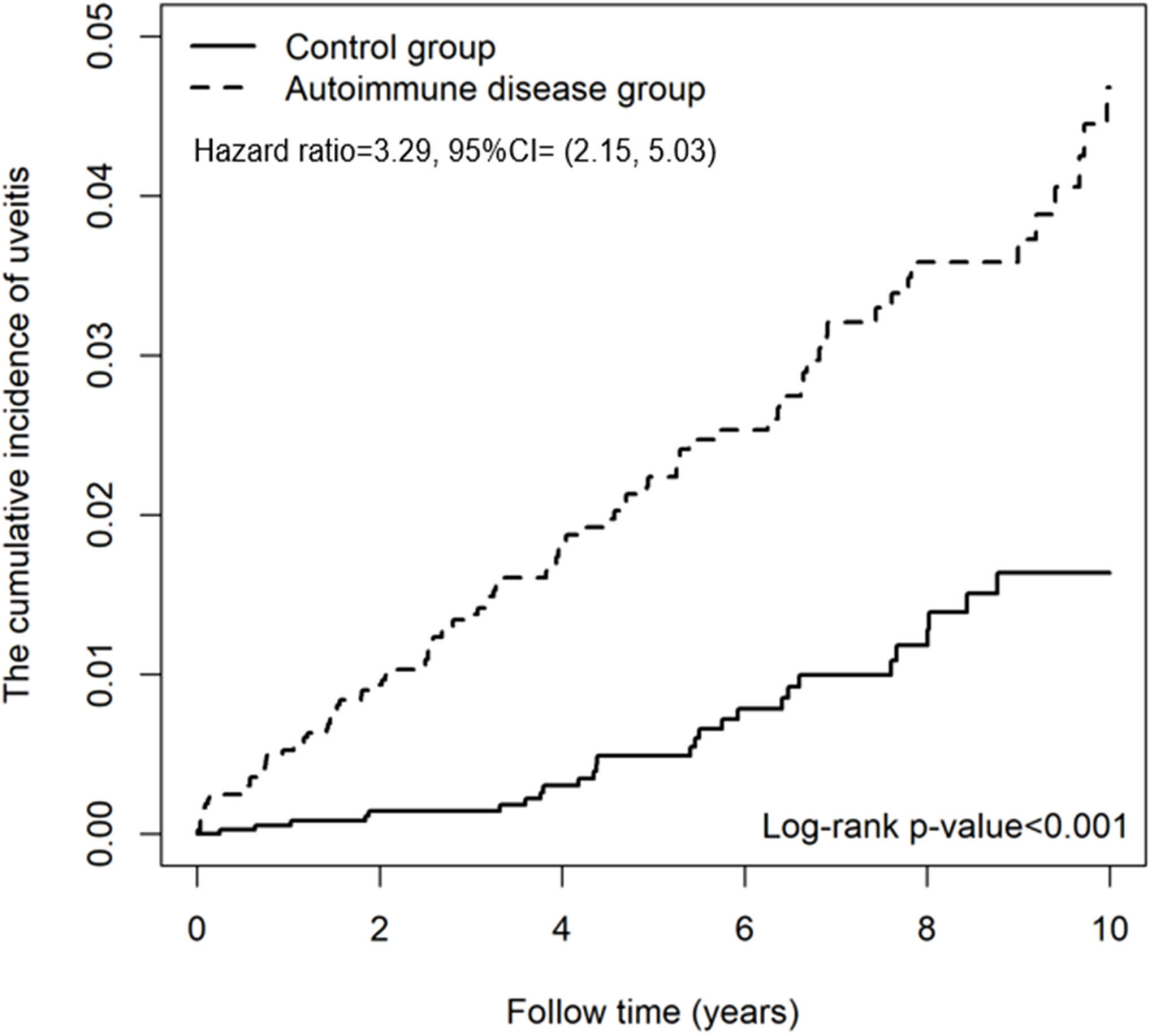
Kaplan–Meier curves for the cumulative incidence of uveitis in children with and without autoimmune diseases. Kaplan–Meier curves comparing the cumulative incidence of uveitis between children and adolescents with autoimmune diseases and matched controls without autoimmune diseases. Patients were followed from the index date until a diagnosis of uveitis, withdrawal, death, or the end of 2020.

In multivariable Cox proportional hazards analysis (**Supplementary Table 5**), patients with autoimmune diseases were associated with increased uveitis risk compared with those without (adjusted hazard ratio [aHR] = 2.65 [95% CI, 1.67-4.19]; P <.001), with significantly different cumulative incidence curves between groups during the 10-year follow-up period (**Figure 2**). Age emerged as an independent risk factor, with higher risk in children aged 10-14 years (aHR = 2.42 [95% CI, 1.43-4.12]; P = .001) and 15-18 years (aHR = 2.27 [95% CI, 1.31-3.93]; P = .004) compared with those younger than 10 years. Among comorbidities, diabetes was independently associated with increased uveitis risk (aHR = 2.34 [95% CI, 1.45-3.78]; P <.001), while lipid metabolism disorders (aHR = 1.76 [95% CI, 0.99-3.13]; P = .06) and hypertension (aHR = 0.81 [95% CI, 0.20-3.37]; P = .77) were not (**Supplementary Table 5**).

Specific autoimmune conditions showed varying degrees of risk (**Supplementary Table 6**), with the highest risk observed in juvenile idiopathic arthritis (aHR = 25.70 [95% CI, 7.41-89.22]; P <.001), followed by rheumatoid arthritis (aHR = 19.70 [95% CI, 5.30-73.25]; P <.001), dermatomyositis/polymyositis (aHR = 19.20 [95% CI, 3.33-111.29]; P <.001), systemic vasculitis (aHR = 16.10 [95% CI, 4.56-57.21]; P <.001), and systemic lupus erythematosus (aHR = 6.41 [95% CI, 1.92-21.37]; P = .003). However, wide confidence intervals suggest these findings should be interpreted with caution due to potential sample size limitations.

### 3.3 Stratified analysis of uveitis risk

In stratified analyses (**Table 2**; **Supplementary Figure 1**), autoimmune diseases were associated with increased uveitis risk in both females (aHR = 2.49 [95% CI, 1.38-4.50]; P = .002) and males (aHR = 2.91 [95% CI, 1.38-6.12]; P = .01). Age-stratified analyses revealed differential risk patterns. While the crude association was significant in children younger than 10 years (crude hazard ratio [cHR] = 4.11 [95% CI, 1.36-12.41]; P = .01), it became nonsignificant after adjustment (aHR = 0.98 [95% CI, 0.23-4.28]; P = .98). However, the association remained significant in children aged 10-14 years (aHR = 2.58 [95% CI, 1.29-5.14]; P = .01) and 15-18 years (aHR = 2.60 [95% CI, 1.27-5.31]; P = .01).

**Table 2:** Stratified analysis of uveitis risk in children with and without autoimmune diseases.

Stratification variables	Autoimmune diseases	Events/PY (IR^†^)	Crude HR (95% CI)	P-Value	Adjusted HR^‡^ (95% CI)	P-Value
Gender						
Female	No	18/11,760 (1.53)	1.00 (Reference)	—	1.00 (Reference)	—
	Yes	45/11,400 (3.95)	2.58 (1.5-4.46)	<.001^***^	2.49 (1.38-4.50)	.002^**^
Male	No	10/8,931 (1.12)	1.00 (Reference)	—	1.00 (Reference)	—
	Yes	44/8,622 (5.1)	4.54 (2.29-9.02)	<.001^***^	2.91 (1.38-6.12)	.01^**^
Age Groups, y						
<10	No	4/7,492 (0.53)	1.00 (Reference)	—	1.00 (Reference)	—
	Yes	15/6,804 (2.2)	4.11 (1.36-12.41)	.01^*^	0.98 (0.23-4.28)	.98
10-14	No	12/6,793 (1.77)	1.00 (Reference)	—	1.00 (Reference)	—
	Yes	42/7000 (6)	3.31 (1.74-6.29)	<.001^***^	2.58 (1.29-5.14)	.01^**^
15-18	No	12/6,407 (1.87)	1.00 (Reference)	—	1.00 (Reference)	—
	Yes	32/6218 (5.15)	2.74 (1.41-5.32)	.003^**^	2.60 (1.27-5.31)	.01^**^
Comorbidities						
Lipid Metabolism Disorders						
No	No	23/19,243 (1.20)	1.00 (Reference)	—	1.00 (Reference)	—
	Yes	79/19,080 (4.14)	3.46 (2.17-5.50)	<.001^***^	2.50 (1.51-4.16)	<.001^***^
Yes	No	5/1,449 (3.45)	1.00 (Reference)	—	1.00 (Reference)	—
	Yes	10/942 (10.62)	3.13 (1.07-9.17)	.04^*^	3.09 (0.99-9.57)	.05
Diabetes						
No	No	5/12,475 (0.40)	1.00 (Reference)	—	1.00 (Reference)	—
	Yes	51/12,037 (4.24)	10.52 (4.20-26.35)	<.001^***^	6.88 (2.54-18.61)	<.001^***^
Yes	No	23/8,217 (2.80)	1.00 (Reference)	—	1.00 (Reference)	—
	Yes	38/7,985 (4.76)	1.70 (1.01-2.85)	.05^*^	1.67 (0.98-2.82)	.06

PY, person-years; IR, incidence rate; HR, hazard ratio; CI, confidence interval.

—Indicates not applicable.

*P <.05; ** P <.01; *** P <.001.

†IR indicates an incidence rate per 1,000 person-years.

‡Adjusted for age, sex, and other medications.

Comorbidity stratification revealed effect modification. Among patients without diabetes, autoimmune diseases were strongly associated with uveitis risk (aHR = 6.88 [95% CI, 2.54-18.61]; P <.001), while the association was attenuated in diabetic patients (aHR = 1.67 [95% CI, 0.98-2.82]; P = .06). Similarly, the association was significant in patients without lipid disorders (aHR = 2.50 [95% CI, 1.51-4.16]; P <.001) but not in those with lipid disorders (aHR = 3.09 [95% CI, 0.99-9.57]; P = .05).

### 3.4 Medication effects on uveitis risk

In analysis of medication use among patients with autoimmune diseases (**Table 3**; **Supplementary Figure 2**), sulfasalazine showed increased uveitis risk in both crude analysis (cHR = 2.48 [95% CI, 1.49-4.12]; P <.001) and after adjustment (aHR = 2.00 [95% CI, 1.04-3.84]; P = .04). Methotrexate demonstrated increased risk in crude analysis (cHR = 1.86 [95% CI, 1.16-2.97]; P = .01) that became nonsignificant after adjustment (aHR = 1.63 [95% CI, 0.87-3.04]; P = .12). Other immunosuppressive agents showed no significant associations in either crude or adjusted analyses: azathioprine (aHR = 0.85 [95% CI, 0.45-1.59]; P = .61), cyclosporine (aHR = 0.87 [95% CI, 0.33-2.27]; P = .77), and mycophenolate (aHR = 0.93 [95% CI, 0.21-4.14]; P = .93).

**Table 3:** Associations between immunosuppressive medication use and uveitis risk in pediatric autoimmune disease patients.

Medication use	Events/PY (IR^†^)	Crude HR (95% CI)	*p*-value	Adjusted HR^‡^ (95% CI)	*p*-value
Corticosteroids					
Prednisolone					
No	60/14320 (4.19)	1.00 (reference)	—	1.00 (reference)	—
Yes	29/5702 (5.09)	1.22 (0.78, 1.9)	.38	1.54 (0.88, 2.7)	.13
Disease-Modifying Medications					
Methotrexate					
No	65/16679 (3.90)	1.00 (reference)	—	1.00 (reference)	—
Yes	24/3343 (7.18)	1.86 (1.16, 2.97)	.01^**^	1.63 (0.87, 3.04)	.12
Sulfasalazine					
No	70/18020 (3.88)	1.00 (reference)	—	1.00 (reference)	—
Yes	19/2002 (9.49)	2.48 (1.49, 4.12)	<.001^***^	2.00 (1.04, 3.84)	.04^*^
Immunosuppressants					
Azathioprine					
No	73/15705 (4.65)	1.00(reference)	—	1.00 (reference)	—
Yes	16/4317 (3.71)	0.80 (0.47, 1.38)	.43	0.85 (0.45, 1.59)	.61
Cyclosporin					
No	84/18647 (4.5)	1.00 (reference)	—	1.00 (reference)	—
Yes	5/1374 (3.64)	0.81 (0.33, 2.01)	.65	0.87 (0.33, 2.27)	.77
Mycophenolate					
No§	-/- (4.48)	1.00 (reference)	—	1.00 (reference)	—
Yes§	-/- (3.29)	0.75 (0.18, 3.03)	.68	0.93 (0.21, 4.14)	.93

PY, person-years; IR, incidence rate; HR, hazard ratio; CI, confidence interval.

—Indicates not applicable.

*P <.05; ** P <.01; *** P <.001.

†IR indicates an incidence rate per 1,000 person-years.

‡Adjusted for age, sex, and other medications.

§Complete event/PY data not available; only IR reported.

Prednisolone effects varied by dosing patterns (**Supplementary Table 7**). In daily dose analysis, high-dose exposure (≥30 mg/day) showed increased risk compared with nonuse (aHR = 2.25 [95% CI, 1.12-4.53]; P = .02) but showed no significant difference compared with low-dose use (aHR = 1.03 [95% CI, 0.46-2.30]; P = .94). In cumulative dose analysis, low-dose exposure (<1740 mg) was associated with increased risk compared with nonuse (aHR = 3.40 [95% CI, 1.85-6.22]; P <.001). When using low dose as reference, moderate cumulative doses (1740-31,355 mg) showed lower risk (aHR = 0.32 [95% CI, 0.13-0.79]; P = .01). High cumulative doses (≥31,355 mg) showed no significant differences compared with either nonuse (aHR = 1.68 [95% CI, 0.73-3.89]; P = .22) or low-dose use (aHR = 0.44 [95% CI, 0.18-1.07]; P = .07).

## 4 Discussion

In this population cohort study of 7,286 matched pediatric patients in Taiwan, autoimmune diseases were associated with a 2.65-fold increased risk of uveitis during a mean follow-up of 5.5 years. The risk varied substantially by age and underlying conditions: while no significant association was observed in children younger than 10 years, the risk was significantly elevated in adolescents aged 10-18 years. Among specific conditions, juvenile idiopathic arthritis showed the strongest association (aHR = 25.70), followed by rheumatoid arthritis and dermatomyositis/polymyositis. Notably, the association between autoimmune diseases and uveitis was modified by metabolic comorbidities, being substantially stronger in patients without diabetes (aHR = 6.88) compared to those with diabetes. Among medications, sulfasalazine was associated with a 2-fold increased risk of uveitis, while other immunosuppressive agents showed no significant associations. Prednisolone effects were dose-dependent: high daily doses (≥30 mg/day) increased risk, while moderate cumulative doses may show potential protective effects compared to low-dose exposure. The study’s strengths include its population-based design using Taiwan’s National Health Insurance Research Database, comprehensive propensity score matching, and detailed analysis of both disease-specific and medication-related risks. These findings provide evidence-based guidance for risk stratification and monitoring strategies in pediatric autoimmune diseases.

The link between autoimmune diseases and uveitis is driven by complex immunopathogenic mechanisms (3, 8, 23). Current evidence points to shared immune dysregulation pathways targeting ocular self-antigens, primarily mediated through T-cell responses and compromised immune tolerance (24–27). Experimental models have demonstrated that activated antigen-specific CD4+ T cells, particularly Th1 and Th17 subsets, disrupt ocular immune privilege by compromising the blood-retinal barrier, leading to persistent intraocular inflammation (24, 28). This mechanism is especially relevant in conditions such as juvenile idiopathic arthritis (JIA)-associated uveitis and Vogt-Koyanagi-Harada (VKH) disease, where cytokine-driven tissue damage results in significant visual morbidity (29, 30).

Our findings suggest roles for population-specific genetic and environmental factors. Prior studies have implicated HLA-DRB1 and PDCD1 polymorphisms in immune-mediated uveitis, with distribution patterns varying by ethnicity (13, 31, 32). Environmental triggers such as infection may also initiate autoimmunity through molecular mimicry or activation of innate immune pathways (33, 34). While gender was not a significant risk factor in our study, consistent with findings from other geographic regions (35–38), the age-dependent susceptibility patterns suggest a crucial role for cumulative environmental exposures in disease development.

Although autoimmune diseases typically first manifest between ages 2-5, the age-stratified risk distribution observed in our study describes a secondary peak in uveitis onset during mid-adolescence, which aligns with existing literature (39–41). This pattern is further supported by regional studies with median onset ages ranging from 9 to 13 years (36, 38, 42, 43). Our findings parallel a previous Taiwan-based study reporting a mean uveitis onset age of 13.4 ± 5.1 years in JIA patients, the leading autoimmune cause of uveitis in adolescents (44). These data highlight the need for age-specific surveillance protocols.

Regarding autoimmune disease distribution, T1DM was the most prevalent autoimmune diagnosis in our cohort (39.39%), followed by SLE (20.2%). This distribution aligns with previous epidemiological studies of pediatric autoimmune diseases in Taiwan (45, 46). For example, JIA predominates in Japan, while autoimmune thyroid disease and celiac disease rank second in Finland and the UK, respectively (47–49). These discrepancies underscore distinct patterns between East Asian and Western populations.

Subgroup analyses revealed that not all autoimmune diseases confer equal risk. SLE, systemic vasculitis, JIA, RA, and DM/PM were each independently associated with elevated uveitis risk. These findings confirm prior reports linking JIA and SLE with ocular inflammation and expand on emerging literature documenting ocular involvement in systemic vasculitides and connective tissue diseases (7, 50). While RA is more commonly associated with adult uveitis, our results suggest possible relevance in pediatric overlap syndromes or misclassified early-onset RA, particularly RF-positive polyarthritis (51). Although data on DM/PM-related uveitis are limited, case studies have reported retinal and vasculitic complications (52–54). However, it must be noted that the wide confidence intervals, especially for less common conditions like DM/PM, reflect small subgroup sample sizes, limiting statistical precision and carrying a risk of overestimation. These findings require validation in larger cohorts.

We also observed variability in uveitis risk based on comorbidities. Notably, autoimmune diseases conferred increased uveitis risk only among patients without diabetes or lipid disorders, whereas this association was not observed among those with these comorbidities. These findings may reflect potential effect modification. Poor glycemic control is a significant and modifiable risk factor for uveitis, with evidence of a dose-response relationship: higher HbA1c levels are associated with increased uveitis incidence, particularly among individuals with type 1 diabetes. This supports the hypothesis that chronic hyperglycemia fosters a pro-inflammatory environment that may initiate or amplify intraocular inflammation (55). Similarly, dyslipidemia has been linked to altered vascular and inflammatory pathways in uveitis (56). Abnormal lipid profiles may promote lipid deposition, oxidative stress, and mitochondrial dysfunction within ocular tissues, thereby amplifying local inflammatory responses and exacerbating tissue damage (57–59)**. T**hese comorbidities may dilute or confound the additional impact of autoimmune disease. It should also be noted that in our “Diabetes - Yes” stratum, the autoimmune group (aHR = 1.67) consists almost exclusively of T1DM patients, while the control group consists of non-autoimmune DM. Therefore, this specific finding should be interpreted as the risk in T1DM relative to other DM types, rather than the added risk of autoimmunity within a general diabetic population. Nonetheless, the absence of a statistically significant association in certain strata may also reflect limited statistical power due to smaller subgroup sizes. The medication analysis revealed distinct relationships between treatment regimens for autoimmune diseases and uveitis risk. Among the agents evaluated, sulfasalazine was the only drug significantly associated with increased uveitis risk, which warrants careful interpretation. This association is likely an example of confounding by indication, rather than a direct pharmacological effect of the drug. This reflects its primary use in enthesitis-related arthritis (ERA) and axial spondyloarthropathy, with ERA being the predominant JIA subtype in Taiwan, accounting for approximately 40% of cases, in contrast to its lower prevalence in Western populations (60, 61). The high prevalence of HLA-B27-associated diseases in Asian populations, combined with their inherent predisposition to anterior uveitis, suggests that the increased uveitis risk is attributable to the underlying high-risk disease subtype for which sulfasalazine was prescribed. This interpretation aligns with previous studies establishing strong links between ERA, ankylosing spondylitis, and uveitis incidence, particularly in Asian populations where these subtypes predominate (3, 8, 23). In contrast, other immunosuppressants—methotrexate, azathioprine, cyclosporin, and mycophenolate—were not significantly associated with uveitis risk, though they are often reserved for more severe or refractory disease. This lack of association may also be influenced by similar confounding-by-indication effects, whereby those receiving more aggressive therapy also represent higher-risk patients (9).

Corticosteroid exposure showed a nuanced, non-linear relationship with uveitis risk. Low cumulative doses were associated with increased risk compared to non-use. This complex finding may reflect protopathic bias, where the medication was initiated to treat early, subclinical inflammation that had not yet met the diagnostic criteria for uveitis. Alternatively, it may simply indicate insufficient disease control at these low doses. Conversely, moderate cumulative doses were associated with a lower risk relative to low-dose users. This observation, while intriguing, warrants cautious interpretation and suggests a hypothesis of potential optimal therapeutic window. High cumulative doses, however, were not associated with this lower risk—possibly due to their use in severe or relapsing disease, again illustrating potential confounding by indication. Daily dose analysis mirrored this pattern: only high daily doses were associated with increased risk, which reinforces the interpretation of confounding by indication, as these doses are likely reflecting their use during acute flares or periods of high disease activity. Therefore, our findings may inform future protocols by suggesting that a certain threshold of ongoing prednisolone exposure (or equivalent immunosuppression) may be protective against uveitis, whereas dropping below that threshold is risky. In practice, clinicians might aim to keep a minimal effective steroid dose until other disease-modifying antirheumatic drugs (DMARDs) take full effect, and plan very gradual tapers with built-in eye exams at close intervals post-taper. Our findings support refining uveitis screening guidelines by proposing a dynamic risk-stratification framework that incorporates factors such as current medication exposure, age, and disease duration. Traditional schedules focus on age at arthritis onset, JIA subtype, and ANA status (9). We propose that children tapering off immunosuppressive therapy—especially steroids—may face short-term spikes in uveitis risk and warrant closer monitoring (e.g., more frequent eye exams shortly after tapering). Conversely, patients stable on systemic therapy for over a year may be at lower risk.

Updated recommendations should also account for high-risk but often under-screened groups, such as those with enthesitis-related arthritis (ERA), who tend to present with acute symptomatic uveitis. Annual screening and symptom education in this group may help detect cases that would be missed by conventional, schedule-based exams. By aligning guidelines with these evolving risk profiles, particularly in settings like Taiwan where screening is not yet standardized, we can better allocate ophthalmologic resources and prevent avoidable vision loss.

## 5 Strengths and limitations

Key strengths of our study include its large, nationally representative sample; well-balanced matching; and detailed assessment of medication effects. To our knowledge, this is one of the few population-based studies of pediatric uveitis risk in an Asian cohort, filling a major knowledge gap. Importantly, we incorporated treatment data into risk modeling, offering insights relevant to real-world clinical decision-making.

This study also has several limitations, primarily stemming from its reliance on administrative claims data. First, although we used the catastrophic illness certificate to maximize diagnostic accuracy, the database lacks granular clinical detail essential for risk stratification, such as disease severity, activity scores, specific ophthalmic exam findings, or key laboratory results (e.g., ANA status), which are potential confounders. Second, medication exposure was based on prescription records, which may not accurately reflect patient adherence or actual intake, which may have introduced nondifferential exposure misclassification. Furthermore, Socioeconomic status and residential factors were not included in the matching process, as insurance premium in pediatric subjects often reflects parental or household information and may not accurately represent the child’s own socioeconomic status. In addition, the registered insured or treatment location can change over time, and a single-time-point geographic proxy may misclassify residence. Therefore, residual confounding from unmeasured social or environmental factors cannot be completely excluded.

Another methodological limitation is the definition of corticosteroid dose. In pediatrics, weight-based dosing (mg/kg/day) is the standard; however, patient weight data was unavailable in the NHIRD, making this correction impossible. We were therefore constrained to using absolute daily doses (e.g., ≥30 mg/day) as a proxy for “high dose.” We must emphasize that a 30mg dose represents a vastly different systemic exposure for children of different ages and body weights. Therefore, these specific dose-response findings must be interpreted with extreme caution.

Finally, as noted in our results, some subgroup analyses (e.g., for DM/PM) had small sample sizes. This limited the statistical precision for these estimates, resulting in wide confidence intervals. Due to these unmeasured variables and data constraints, residual confounding cannot be fully excluded.

## 6 Conclusion

In conclusion, pediatric patients with autoimmune diseases—especially those in adolescence or treated with sulfasalazine—face significantly increased risk of uveitis. Our findings support the development of risk-adapted screening protocols and suggest that moderate, sustained corticosteroid therapy may be associated with protective benefits. Additional research should aim to improve uveitis prediction and prevention through biomarker discovery and advanced risk modeling. Genetic markers beyond ANA and HLA-B27, such as specific HLA alleles, cytokine profiles, may offer earlier and more precise risk stratification. Ultimately, these efforts—combined with longitudinal cohort studies and targeted trials—can enable precision medicine approaches that identify high-risk children early and tailor interventions to prevent vision-threatening disease.

## Data Availability

The raw data supporting the conclusions of this article will be made available by the authors, without undue reservation.
