# Supplementary material for: Risk of uveitis among children with autoimmune diseases: a nationwide matched-cohort study of 3,643 cases
Source: Front Immunol. 2025 Dec 4;16:1717805. doi: 10.3389/fimmu.2025.1717805 (PMC12711771; doi:10.3389/fimmu.2025.1717805)
Supplement: Supplementary file 1 [file Table1.docx]

Supplementary Material

# Supplementary Data

## Supplementary Method 1.

Exclusion criteria: (1) Patients with the year of index before 2009 and after 2019. (2) Patients who were diagnosed with uveitis before the index date. (3) Patients age >18 years old. (4) Patients who were diagnosed with underlying disease of viral hepatitis (ICD-9-CM: 070, V02.61, V02.62; ICD-10-CM: B15-B19, Z22.51, Z22.52), human immunodeficiency virus infection (ICD-9-CM: 042-044, 795.8, V08; ICD-10-CM: B20, Z21, Z22.6), TB (ICD-9-CM: 010-018; ICD-10-CM: A15, A17-A19) before the index date.

# Supplementary Figures and Tables

## Supplementary Figures

## Supplementary Figure 1. Forest Plot of Stratified Analysis of Uveitis Risk in Children With and Without Autoimmune Diseases.


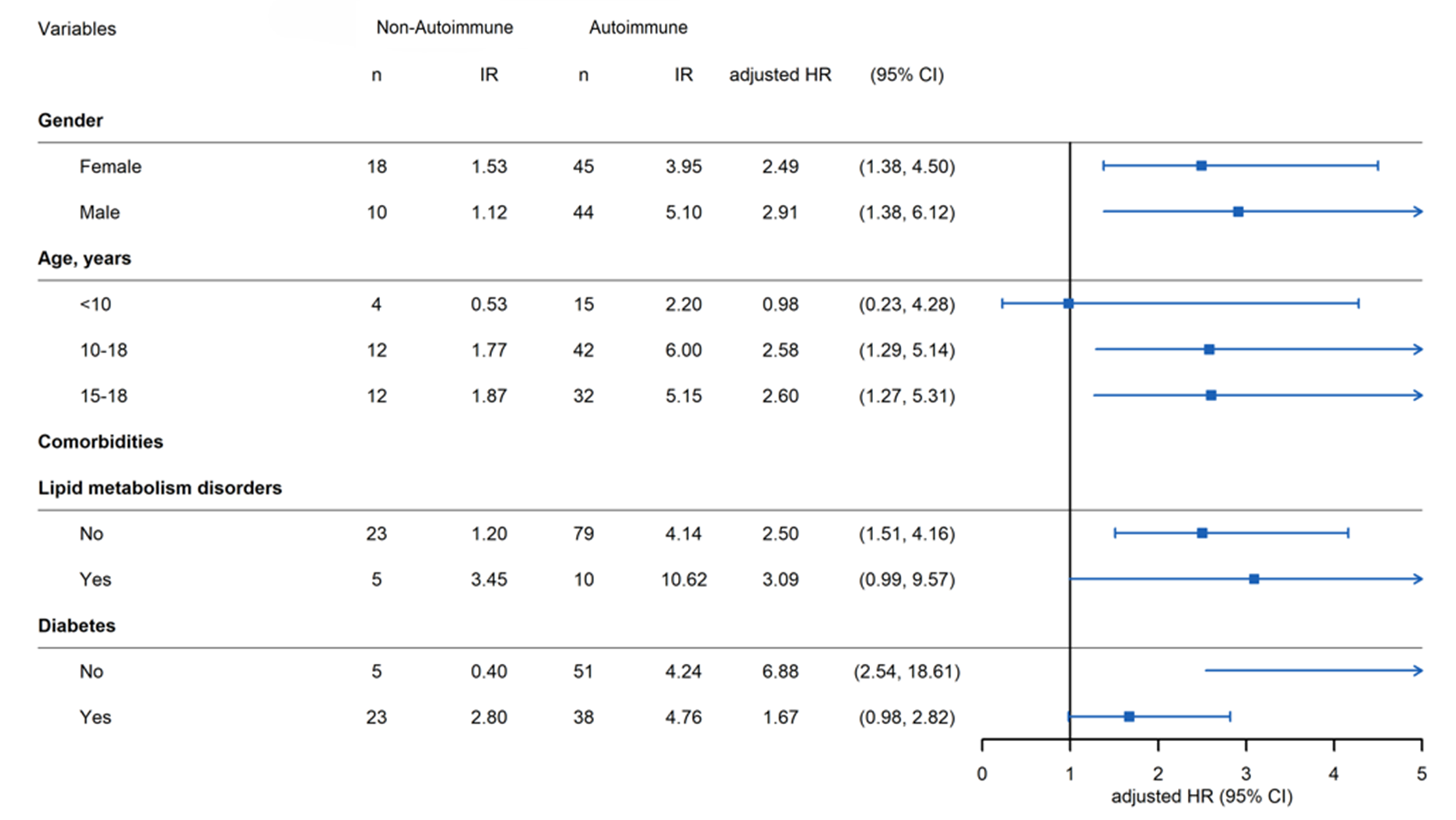


Forest plot showing adjusted hazard ratios for uveitis in patients with autoimmune diseases, stratified by sex, age group (<10, 10–14, and 15–18 years), and the presence of comorbidities including diabetes, and lipid metabolism disorders. The Cox regression models were adjusted for demographic and clinical covariates.

Abbreviations: aHR, adjusted hazard ratio; CI, confidence interval.

**Supplementary Figure 2. Forest Plot of Associations Between Immunosuppressive Medication Use and Uveitis Risk in Pediatric Autoimmune Disease Patients.**


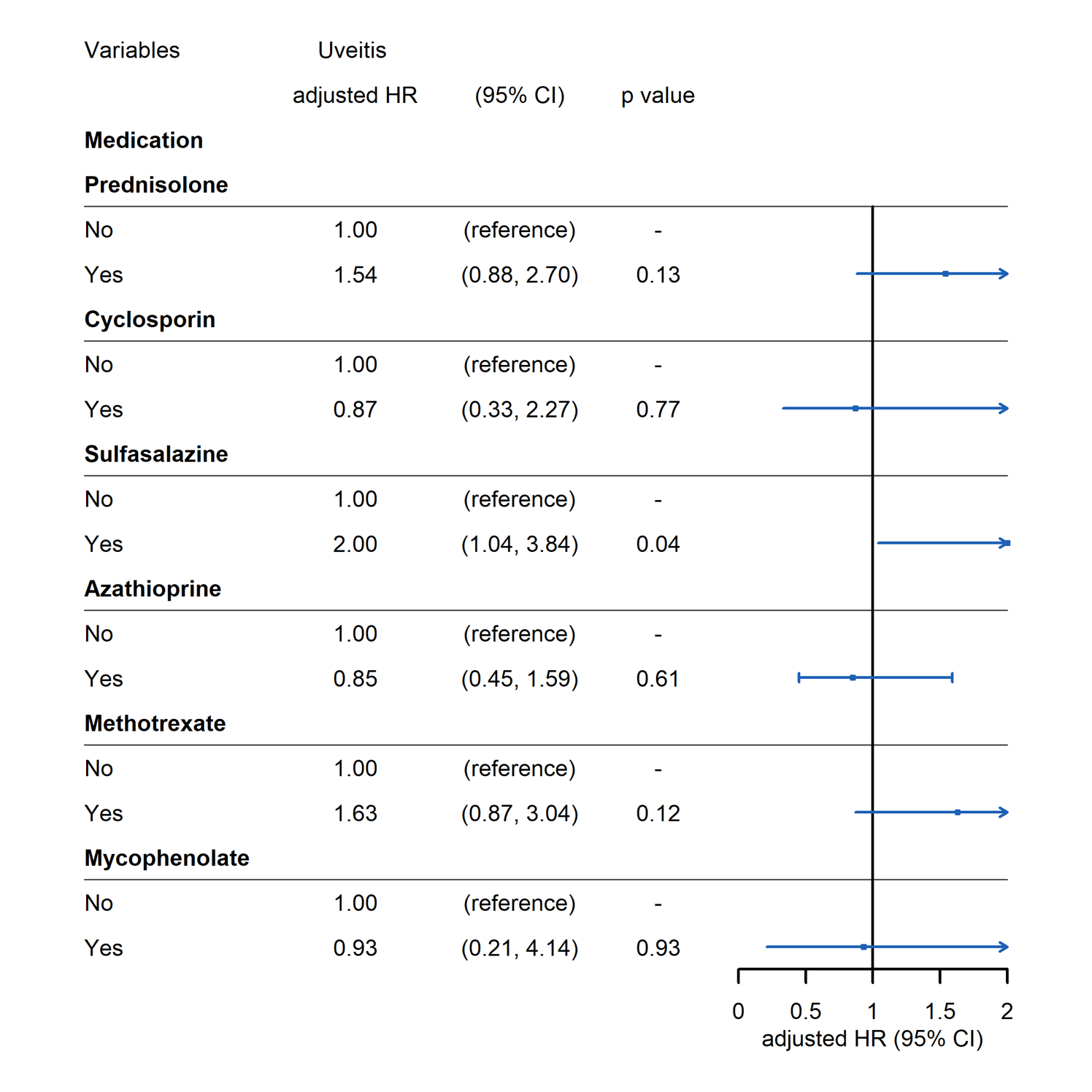


Forest plot showing adjusted hazard ratios for uveitis associated with the use of immunosuppressive medications, including prednisolone, methotrexate, sulfasalazine, azathioprine, cyclosporine, and mycophenolate. Models were adjusted for demographic and clinical covariates.

Abbreviations: aHR, adjusted hazard ratio; CI, confidence interval.

## Supplementary tables

**Supplementary table 1. ICD-9 and ICD-10 Codes Used to Define Autoimmune Diseases.**

| **Autoimmune diseases** | **ICD-9-CM Codes** | **ICD-10-CM Codes** |
| --- | --- | --- |
| Juvenile idiopathic arthritis | 714.3x | M08 |
| Rheumatoid arthritis | 714.0-714.2 | M05, M06 |
| Systemic lupus erythematosus | 710.0 | M32 |
| Sjogren’s syndrome | 710.2 | M35.0 |
| Dermatomyositis/Polymyositis | 710.3, 710.4 | M33 |
| Systemic vasculitis | 136.1, 446.0-446.2, 446.4, 446.7, 447.6 | M30.1, M30.3, M30.8, M31.4, M31.7, M35.2 |
| Type 1 diabetes mellitus | 250.01, 250.03, 250.11, 250.13, 250.21, 250.23, 250.31, 250.33, 250.41, 250.43, 250.51, 250.53, 250.61, 250.63, 250.71, 250.73, 250.81, 250.83, 250.91, 250.93 | E10 |
| Autoimmune thyroid disease | 242.0, 245.2 | E05.0, E06.3 |
| Ankylosing spondylitis | 720.0 | M45 |
| Inflammatory bowel disease | 555, 556.9 | K50, K51 |
| Psoriatic arthritis | 696.0 | L40.5x |
| Multiple sclerosis | 340 | G35 |
| Myasthenia gravis | 358.0 | G70.0 |
| Autoimmune hemolytic anemia | 283.0 | D59.0, D59.1, D59.3-D59.6, D59.8, D59.9 |
| Immune thrombocytopenic purpura | 287.3 | D69.3 |
| Antiphospholipid syndrome | 289.8 | D68.61 |
| Autoimmune hepatitis | 571.49 | K75.4 |

**Supplementary table 2. ICD-9-CM and ICD-10-CM Codes Used to Define Uveitis and Comorbidities.**

| **Conditions** | **ICD-9-CM Codes** | **ICD-10-CM Codes** |
| --- | --- | --- |
| Uveitis | 360.00, 360.11, 360.12, 362.18, 363.00, 363.01, 363.03, 363.05, 363.08, 363.1, 363.20, 363.21, 363.4, 364.00, 364.02, 364.04, 364.1, 364.3 | H20.00, H20.02, H20.04, H20.1, H20.9, H30.00-H30.03, H30.1, H30.2, H30.89, H30.9, H31.1, H35.06, H44.00, H44.11, H44.13 |
| Lipid metabolism disorders | 272 | E71.30, E75.21, E75.22, E75.24, E75.3, E75.5, E75.6, E77, E78.0-E78.70, E78.79, E78.8, E78.9 |
| Hypertension | 401-405 | I10-I13, I15, N26.2 |
| Diabetes mellitus | 250 | E08-E13 |

**Supplementary table 3. ATC Codes for Medications Assessed.**

| **Medication** | **ATC Codes** |
| --- | --- |
| Prednisolone | H02AB04, H02AB06 |
| Sulfasalazine | A07EC01 |
| Azathioprine | L04AX01 |
| Methotrexate | L04AX03 |
| Mycophenolate | L04AA06 |
| Cyclosporin | L04AD01 |

**Supplementary table 4. Frequency Distribution of Autoimmune Diseases Among Pediatric Patients in the Study Cohort.**

| **Autoimmune disease** | **No. (%) ^*^** |
| --- | --- |
| Type 1 diabetes mellitus | 1435 (39.4) |
| Systemic lupus erythematosus | 736 (20.2) |
| Systemic vasculitis | 410 (11.3) |
| Juvenile idiopathic arthritis | 336 (9.2) |
| Rheumatoid arthritis | 219 (6.0) |
| Myasthenia gravis | 204 (5.6) |
| Inflammatory bowel disease | 147 (4.0) |
| Dermatomyositis/Polymyositis | 52 (1.4) |
| Sjogren’s syndrome | 40 (1.1) |
| Multiple sclerosis | 38 (1.0) |
| Systemic sclerosis | 9 (0.3) |
| Others | 17 (0.5) |

* Data is presented as No. (%). N = 3643 participants and percentages may not sum to 100% due to rounding.

**Supplementary table 5. Risk Factor Analyses for Uveitis Among All Study Individuals.**

| **Risk Factors** | Events/PY | IR^†^ | Crude HR (95% CI) | P-Value | Adjusted HR^‡^ (95% CI) | P-Value |
| --- | --- | --- | --- | --- | --- | --- |
| **Primary Exposure** | | | | | | |
| Autoimmune diseases | | | | | | |
| No | 28/20691 | 1.35 | 1.00 (reference) | — | 1.00 (reference) | — |
| Yes | 89/20022 | 4.45 | 3.29 (2.15, 5.03) | <.001^***^ | 2.65 (1.67, 4.19) | <.001^***^ |
| **Demographic Factors** | | | | | | |
| Gender | | | | | | |
| Female | 63/23160 | 2.72 | 1.00 (reference) | — | 1.00 (reference) | — |
| Male | 54/17554 | 3.08 | 1.14 (0.79, 1.63) | .49 | 1.05 (0.72, 1.53) | .81 |
| Age groups, y | | | | | | |
| <10 | 19/14295 | 1.33 | 1.00 (reference) | — | 1.00 (reference) | — |
| 10-14 | 54/13793 | 3.92 | 2.92 (1.73, 4.92) | <.001^***^ | 2.42 (1.43, 4.12) | .001^**^ |
| 15-18 | 44/12624 | 3.49 | 2.59 (1.51, 4.44) | <.001^***^ | 2.27 (1.31, 3.93) | .004^**^ |
| **Comorbidities** | | | | | | |
| Lipid metabolism disorders | | | | | | |
| No | 102/38322 | 2.66 | 1.00 (reference) | — | 1.00 (reference) | — |
| Yes | 15/2391 | 6.27 | 2.36 (1.37, 4.05) | .002^**^ | 1.76 (0.99, 3.13) | .06 |
| Hypertension | | | | | | |
| No^§^ | NA/NA | 2.88 | 1.00 (reference) | — | 1.00 (reference) | — |
| Yes^§^ | NA/NA | 2.64 | 0.92 (0.23, 3.71) | .90 | 0.81 (0.20, 3.37) | .77 |
| Diabetes | | | | | | |
| No | 56/24511 | 2.28 | 1.00 (reference) | — | 1.00 (reference) | — |
| Yes | 61/16202 | 3.76 | 1.64 (1.14, 2.35) | .01^**^ | 2.34 (1.45, 3.78) | <.001^***^ |

Abbreviations: PY, person-years; IR, incidence rate; HR, hazard ratio; CI, confidence interval.

— Indicates not applicable.

* P < .05; ** P < .01; *** P < .001.

† IR indicates an incidence rate per 1,000 person-years.

‡ Adjusted for age, sex, and other medications.

§ Complete event/PY data not available; only IR reported.

**Supplementary table 6. Risk Factor Analyses for Uveitis Among All Study Individuals in Different Types of Autoimmune Diseases.**

| **Autoimmune diseases** | Events/PY (IR^†^) | Crude HR (95% CI) | P-Value | Adjusted HR^‡^ (95% CI) | P-Value |
| --- | --- | --- | --- | --- | --- |
| None | 28/20691 (1.35) | 1.00 (reference) | — | 1.00 (reference) | — |
| Type 1 diabetes mellitus | 37/8084 (4.58) | 3.36 (2.06, 5.49) | <.001^***^ | 1.63 (0.97, 2.74) | .06 |
| Systemic lupus erythematosus | 6/4117 (1.46) | 2.51 (1.32, 4.76) | .01^**^ | 6.41 (1.92, 21.37) | .003^**^ |
| Systemic vasculitis | 6/1560 (3.85) | 3.04 (1.25, 7.39) | .01^*^ | 16.10 (4.56, 57.21) | <.001^***^ |
| Juvenile idiopathic arthritis | 14/1715 (8.16) | 6.92 (3.74, 12.79) | <.001^***^ | 25.70 (7.41, 89.22) | <.001^***^ |
| Rheumatoid arthritis^§^ | -/- (0.65) | 5.64 (2.86, 11.09) | <.001^***^ | 19.70 (5.3, 73.25) | <.001^***^ |
| Myasthenia gravis^\|\|^ | 16/1388 (11.53) | NA | .98 | NA | .98 |
| Inflammatory bowel disease^§^ | -/- (1.46) | 1.11 (0.15, 8.17) | .92 | 3.29 (0.36, 30.13) | .29 |
| Dermatomyositis/Polymyositis | 6/325 (18.46) | 4.49 (1.07, 18.84) | .04^*^ | 19.20 (3.33, 111.29) | <.001^***^ |
| Sjogren’s syndrome | 6/250 (24.00) | 2.92 (0.4, 21.48) | .29 | 8.28 (0.93, 73.58) | .06 |
| Multiple sclerosis^\|\|^ | 12/225 (53.33) | NA | .99 | NA | .99 |
| Systemic sclerosis^§\|\|^ | -/- (55.56) | NA | >.99 | NA | >.99 |
| Others^\|\|^ | 6/89 (67.42) | NA | .99 | NA | .99 |

Abbreviations: NA, not applicable; PY, person-years; IR, incidence rate; HR, hazard ratio; CI, confidence interval.

— Indicates not applicable.

* P < .05; ** P < .01; *** P < .001.

† IR indicates an incidence rate per 1,000 person-years.

‡ Adjusted for age, sex, and other medications.

§ Complete event/PY data not available; only IR reported.

|| Complete hazard ratio data not available.

**Supplementary table 7. Dose-Dependent Effects of Prednisolone on Uveitis Risk Among Children With Autoimmune Diseases.**

| **Prednisolone dose** | Events/PY (IR^†^) | Adjusted HR^‡^ vs Non-user (95% CI) | P-Value | Adjusted HR^‡^ vs Low dose user (95% CI) | P-Value |
| --- | --- | --- | --- | --- | --- |
| **Daily dose, mg** | | | | | |
| Non-user (N=6170) | 87/34583 (2.52) | 1.00 (reference) | — | — | — |
| Low dose (< 7.5) (N=469) | 13/2665 (4.88) | 1.90 (0.95, 3.79) | .07 | 1.00 (reference) | — |
| Moderate dose (7.5-30) (N=164) | 5/767 (6.52) | 2.50 (0.96, 6.5) | .06 | 1.13 (0.39, 3.24) | .83 |
| High dose (≥ 30) (N=483) | 12/2698 (4.45) | 2.25 (1.12, 4.53) | .02^*^ | 1.03 (0.46, 2.3) | .94 |
| **Cumulative dose, mg** | | | | | |
| Non-user (N=6170) | 87/34583 (2.52) | 1.00 (reference) | — | — | — |
| Low dose (< 1740) (N=337) | 15/1649 (9.10) | 3.40 (1.85, 6.22) | <.001^***^ | 1.00 (reference) | — |
| Moderate dose (1740-31355) (N=399) | 7/2233 (3.13) | 1.16 (0.49, 2.75) | .74 | 0.32 (0.13, 0.79) | .01^*^ |
| High dose (≥ 31355) (N=380) | 8/2248 (3.56) | 1.68 (0.73, 3.89) | .22 | 0.44 (0.18, 1.07) | .07 |

Abbreviations: PY, person-years; IR, incidence rate; HR, hazard ratio; CI, confidence interval.

— Indicates not applicable.

* P < .05; ** P < .01; *** P < .001.

† IR indicates an incidence rate per 1,000 person-years.

‡ Adjusted for age, sex, and other medications.
